# Family Functioning and Internalizing and Externalizing Problems in Gifted Children

**DOI:** 10.3390/ejihpe14050077

**Published:** 2024-04-27

**Authors:** Maria Assunta Zanetti, Tommaso Trombetta, Luca Rollè, Carlo Marinoni

**Affiliations:** 1 Department of Brain and Behavioral Sciences, University of Pavia, 27100 Pavia, Italy; zanetti@unipv.it (M.A.Z.); carlo.marinoni01@universitadipavia.it (C.M.); 2 Department of Psychology, University of Turin, 10124 Torino, Italy; l.rolle@unito.it

**Keywords:** gifted children, child behavior checklist, internalizing problems, externalizing problems, family functioning, FACES-IV

## Abstract

Introduction: Although gifted children can express high cognitive skills, they can also show socioemotional difficulties. Drawing from Olson’s circumplex model, the present paper assessed the role of family functioning in internalizing and externalizing problems in gifted children. Materials and Methods: 362 mothers and their 362 gifted children were included. The unbalanced subscales of the FACES IV—disengagement, enmeshment, rigidity, and chaos—and the CBCL were administered to mothers. The children completed the WISC-IV. Results: The model predicting internalizing problems was significant and explained 5.6% of the variance. Only rigidity had an independent and positive effect on internalizing problems in gifted children over and above sociodemographic variables and QI, whereas disengagement, enmeshment, and chaos were not associated with internalizing problems. The model predicting externalizing problems was significant and explained 10% of the variance. Again, rigidity was the only variable that had an independent and positive effect on externalizing problems in gifted children over and above sociodemographic variables and QI, whereas disengagement, enmeshment, and chaos were not associated with externalizing problems in this population. Discussion: Rigid families with a low ability to change in conditions that require readjustment appear to increase the risk of both internalizing and externalizing problems in gifted children. Although further studies are needed to support these preliminary findings, parental support interventions aimed at increasing flexibility appear to be useful.

## 1. Introduction

The majority of authors agree in identifying giftedness in those individuals who, compared to their peers, have the potential to demonstrate exceptional abilities in one or more areas: general intellectual abilities, specific aptitudes or creative thinking, which leads them on a cognitive level to reach earlier than typical development times high reasoning skills not always in line with the level of emotional development [1,2,3,4,5,6]. This condition can expose them to greater emotional dysregulation, resulting in maladaptive behaviors, both internalizing and externalizing, which may be influenced by life and family contexts [7,8].

This phenomenon is poorly studied, especially in Italy, and there are stereotypes about giftedness, such as the assumption that possessing a high cognitive potential is a guarantee of success in the individual’s educational and life path [9]. In contrast, gifted children can also show difficulties in recognition, management and regulation of emotion, fragility in social relational skills, extreme perfectionism, and intense cognitive activation that is difficult to manage at a behavioral level [10]. In addition, giftedness can be accompanied by an asynchronous development of cognitive, socio-emotional, and psychomotor abilities [11] and feelings of inadequacy and misunderstanding due to the weight of expectations [12]. According to these results, exploring risk factors of adjustment and behavioral problems among gifted children seems necessary.

### 1.1. Internalizing and Externalizing Behaviors in Gifted Children

Extensive research has identified two categories of behaviors that comprise most of the variance in children’s behavioral problems, referred to as internalizing and externalizing problems [13,14,15]. Internalizing problems concern children who are often described as depressed, anxious, withdrawn or who have problems that are more internal to the child’s emotional experience. Externalizing problems, on the other hand, concern children who are identified as hyperactive, aggressive, uninhibited, or disruptive in their behaviors.

Internalizing behavioral problems are commonly observed in gifted children, such as anxiety [16], social withdrawal [17], low self-esteem [18] and excessive perfectionism [16]. Children who show internalizing problems can also present externalizing problems such as psychomotor instability, irritability, or aggressive behavior [19]. Some studies have found an association between externalizing problems and giftedness [16,20]. Although the literature on externalizing problems among gifted children is inconsistent, since not all research yielded the same results [21], some researchers have hypothesized that, among gifted children, those with a higher IQ may have more externalizing problems and adaptation difficulties [22]. For example, significant social adjustment difficulties were found in children with the highest IQ in Terman’s famous cohort [23]. According to these findings and considering the adjustment problems that can be observed among gifted children, the present study aimed to assess risk factors of internalizing and externalizing problems in this population, controlling for the confounding effect of IQ.

### 1.2. Family Functioning and Internalizing and Externalizing Problems

Family functioning can play a role in predicting internalizing and externalizing problems, as supported within the general population [24,25,26,27,28]. Olson’s circumplex model of marital and family systems represents a useful framework for understanding family functioning and provides suggestions for interventions [29]. It defines family functioning according to two main dimensions: (1) cohesion, which refers to the degree of closeness among family members; (2) and adaptability, which is the ability of the family system to change based on developmental and environmental demands. Balanced levels of cohesion and adaptability promote positive family functioning. On the other hand, unbalanced levels of cohesion and adaptability (i.e., very low or very high) are related to lower levels of family functioning. The Family Adaptation and Cohesion Evaluation Scale IV (FACES IV) [29,30] is used to assess family functioning according to the circumplex model. It consists of two balanced subscales, cohesion and adaptability, and four unbalanced subscales, which cover the lower and upper end of cohesion—disengaged, enmeshed—and adaptability—rigid and chaotic. Higher levels of cohesion and adaptability reflect better family functioning; in contrast, higher levels of disengagement, enmeshment, rigidity, and chaos correspond to lower family functioning.

Despite the limited literature in this field, some studies supported a link between the dimensions of the circumplex model and internalizing and externalizing problems among children within the general population. In particular, cohesion and adaptability have been found to be related to depression, social anxiety and fear, aggressive and rule-breaking behaviors, bullying, and general indexes of internalizing and externalizing problems [23,24,25,26,31], with cohesion as the dimension more consistently related to internalizing and externalizing problems. However, other studies are needed to assess the influence of family functioning on internalizing and externalizing problems in children, focusing also on the unbalanced dimensions of the FACES-IV, which seem poorly considered in this field.

### 1.3. Family Functioning and Internalizing and Externalizing Problems in Gifted Children

While the relationship between family functioning and internalizing and externalizing problems received some confirmation within the general population, only limited studies have applied Olson’s circumplex model to assess this relation in families with gifted children [32,33]; studies on the role of family functioning in this population have mainly focused on talent development rather than on internalizing and externalizing problems. Nonetheless, preliminary findings on the positive influence of cohesion and adaptability on social competences in gifted children emerged in the study by Olszewski-Kubilius et al. [32]. In addition, some studies have found a relationship between other dimensions of the family environment—parenting style, style of communication, level of conflict, family satisfaction, and parental support and acceptance—and indicators of gifted child adjustment in terms of both internalizing and externalizing problems—anxiety, self-esteem, self-control, conduct problems, prosocial behaviors, negative perfectionism, and overall adjustment [22,32,34]. Although through a curvilinear approach, an analysis of the preliminary data did not identify extreme patterns of family functioning in families with gifted children [33], and several studies found authoritative parenting styles, positive communication, and high levels of parental support among this population [32], other studies are needed to understand the impact of family functioning on children’s internalizing and externalizing problems in this population through a linear approach to explain individual differences in adjustment problems among gifted children [29,35].

Accordingly, using two multiple linear regression models, the current study aims to fill this gap evaluating the influence of unbalanced dimensions of family functioning (i.e., disengagement, enmeshment, rigidity, and chaos) on internalizing and externalizing problems in gifted children, in order to inform services and interventions aimed at improving child and family wellbeing within this specific population.

### 1.4. Hypotheses

**H1:**  *Higher scores on the unbalanced dimensions of family functioning—disengagement, enmeshment, rigidity, and chaos—are associated with higher levels of child’s internalizing problems*.

**H2:**  *Higher scores on the unbalanced dimensions of family functioning—disengagement, enmeshment, rigidity, and chaos—are associated with higher levels of child’s externalizing problems*.

## 2. Materials and Methods

### 2.1. Procedure

The participants came from different regions of northern, central and southern Italy and were assessed by the Italian Laboratory for the Study and Development of Potential, Talent and Giftedness (LabTalento) at the University of Pavia. In this way, it was possible to collect a large sample, heterogeneous in terms of geographical origin (see Table 1), which gives additional strength to the data collected.

The child’s family contacted LabTalento to investigate possible giftedness, which is often associated with relationship difficulties, boredom or learning/behavioral problems, especially in the school context. Data were collected together with a full IQ assessment and analyzed for research purposes. The assessment took place at the University of Pavia and involved both the child and the parents, also using self-report questionnaires. To participate in the study, a) the children had to have an IQ score of 120 or higher, and b) the mothers had to participate in the study. The Ethics Committee of the University of Pavia approved the research project (protocol code 057/20, approved on 21 May 2020). All parents gave informed consent for themselves and their children. The children were also informed about the procedures and the purpose of the study before participating in the research. As only few data were available on fathers and to increase the generalizability of the data emerged to the population of mothers, only the mothers of gifted children were included in the present study.

### 2.2. Participants

The participants were 362 gifted children, aged between 6 and 10 years old (297 males and 65 females; age: M = 8.03; SD = 1.30), and their 362 mothers, aged between 30 and 53 years old (age: M = 42.24; SD = 4.10). The IQ of all children was above the threshold of 120, which is used to classify children as gifted children according to Ruf & Valley [36].

**Figure 1:**
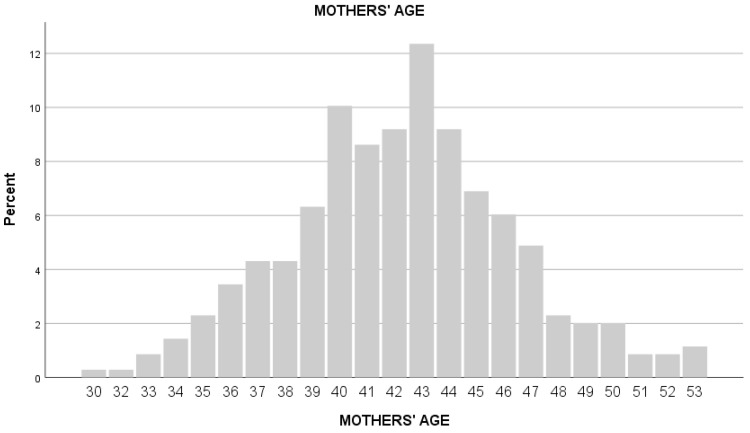
The figure shows the age distribution of the mothers.

**Figure 2:**
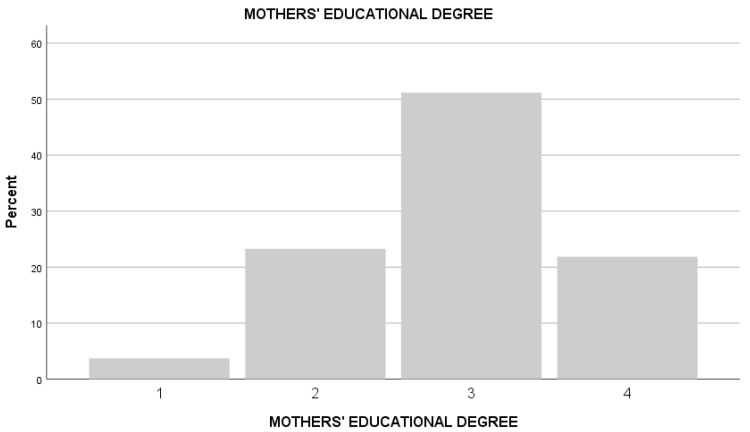
The figure shows the educational degree distribution of the mothers. Note: 1 = Lower secondary degree; 2 = Upper secondary degree; 3 = University degree; 4 = Master post degree.

The presence of a large majority of males compared to females is consistent with the literature in this field, which identified higher levels of acting out among males and greater adaptive capacities among females, which in turn can limit the necessity of a cognitive evaluation [37]. Sociodemographic characteristics of the sample are presented in Table 1, Figure 1 and Figure 2.

### 2.3. Measures

A series of assessment measures were used: children completed the Weschler Intelligence Scale for Children IV (WISC-IV) [37,38]; a sociodemographic schedule, the Family Adaptation and Cohesion Evaluation Scales IV (FACES IV) [29,30,39], and the Child Behavior Checklist (CBCL) [13] were administered to the mothers.

Wechsler Intelligence Scale for Children (WISC-IV) [37,38]: The WISC-IV is one of the most widely used clinical instruments to assess the cognitive abilities of subjects aged between 6 years and 0 months to 16 years and 11 months. It assesses four areas of cognitive functioning: verbal comprehension, visual–perceptual reasoning index, working memory index and processing speed index, which together determine the total IQ score. The Italian version of the WISC-IV was used in the present study [38].

Family Adaptation and Cohesion Evaluation Scale IV (FACES) [29,30]: the Italian version [39] of the FACES IV was used to assess family functioning. The FACES IV is a widely used instrument for assessing family functioning according to Olson’s circumplex model. It has 30 items, which participants respond to using a 5-point Likert scale (from “never” to “always”). It assesses family functioning along two balanced dimensions—cohesion and adaptability (α = 0.72)—and four unbalanced dimensions covering the lower and upper end of cohesion—disengaged (α = 0.71), enmeshed (α = 0.62)—and adaptability—rigid (α = 0.70) and chaotic (α = 0.61). The FACES IV package also contains two additional scales, namely family communication and family satisfaction. Of these eight scales, only disengagement, enmeshment, rigidity, and chaos were used in the present study.

Child Behavioral Checklist (CBCL) [9]: the CBCL is one of the major tools used to assess emotional and behavioral problems in children and adolescents between the ages of 6 and 18. It is filled out by parents and consists of 118 items. The answers are on a 3-point Likert scale and refer to their children’s behavior in the 6 months prior to completion. The CBCL assesses 8 dimensions: “withdrawn” (WI) (α = 0.62), “somatic complaints” (SC) (α = 0.61), “anxious/depressed” (AD) (α = 0.73), “social problems” (SP) (α = 0.61), “thinking problems” (TPs) (α = 0.60), “attention problems” (APs) (α = 0.74), “delinquent behavior” (DB) (α = 0.77) and “aggressive behavior” (AB) (α = 0.78). These dimensions are grouped into two higher-order factors, namely internalizing and externalizing problems. In addition, a total score can be computed, which provides a general index of adjustment problems. For the current study, internalizing and externalizing subscales were considered.

### 2.4. Data Analysis

Descriptive statistics and frequency analyses were used to describe the study sample. As recommended by Tabachnick and Fidell [40], the studied variables were tested for the assumptions of normality. According to the results of the Shapiro–Wilk test, the data on internalizing (W: 0.980; *p* < 0.001) and externalizing problems (W: 0.985; *p* < 0.010) violated the normality condition. Consequently, a two-step process for transforming the dependent variables toward normality was used following Templeton [41]. Preliminary analyses were performed to investigate the bivariate correlation (Pearson’s correlation) between the study variables, and a *t*-test analysis was used to investigate sex differences in IQ. Finally, multiple linear regression analyses were used to test the association between the independent (i.e., disengagement, enmeshment, rigidity, and chaos) and dependent (i.e., children’s internalizing and externalizing problems) variables included in the research design, controlling for the effect of childrens’ sex, age, and IQ.

## 3. Results

The mean score of the children’s IQ was 134.83. There were no significant differences in terms of IQ according to the sex of the children (see Table 2). Bivariate correlations between the study variables are shown in Table 3.

Two multiple linear regression models were performed on the CBCL dimensions. The first regression model was performed to explore the influence of disengagement, enmeshment, rigidity, and chaos on child’s internalizing problems, controlling for the effect of child’s sex, age, and IQ (see Table 4). The model was significant (F(7, 354): 2.535; *p* < 0.05) and explained 5.6% of the variance in internalizing problems (R2: 0.056). Only rigidity (b: 0.15; 95% CI [0.03; 0.21]; *p* < 0.05) was positively associated with internalizing problems in gifted children. No other variables included in the model were significantly associated with internalizing problems.

The second linear regression model assessed the influence of disengagement, enmeshment, rigidity, and chaos on child’s externalizing problems, controlling for the effect of child’s sex, age, and IQ (see Table 5). The model was significant (F(7, 354): 4.733; *p* < 0.001) and explained 10% of the variance in externalizing problems (R2: 0.100). Only rigidity (b: 0.28; 95% CI [0.12; 0.29]; *p* < 0.001) was positively associated with externalizing problems in gifted children. No other variables in the model were significantly associated with externalizing problems.

## 4. Discussion

The current study aimed to assess, according to Olson’s Circumplex Model [29], the impact of family functioning on internalizing and externalizing problems among gifted children, controlling for the confounding effect of sociodemographic variables and child IQ.

Considering the first hypothesis regarding the influence of the unbalanced dimensions of family functioning—disengagement, enmeshment, rigidity, and chaos—on internalizing problems., in the present study, only rigidity increased the risk of internalizing problems in gifted children over and above sociodemographic variables and QI. These data are in partial contrast to the literature on this topic [26,31], which tends to highlight a marginal role of the adaptability dimension in internalizing problems. Moreover, it is worth noting that the lack of a relationship between unbalanced cohesion and internalizing symptoms contrasts with the literature on the general population, which instead tends to show a relationship between these variables [23,24,31]. This seems to suggest that there are pathways leading to internalizing problems that differ depending on the population studied. Further studies investigating the role of family functioning in internalizing problems in gifted children are therefore needed to support and further investigate the preliminary results that emerged here, also including control samples from the general population.

Regarding the second hypothesis on the effect of the unbalanced dimensions of family functioning—disengagement, enmeshment, rigidity, and chaos—on child’s externalizing problems, the present study, again, found that only rigidity (i.e., the lower end of adaptability) positively influenced externalizing problems over and above sociodemographic variables and IQ. These findings are in line with results found both in the general population and among gifted children [22,24,32].

According to the results that emerged in the present study, rigid families with strict and coercive discipline and a low capacity for change in situations that require family readjustment, such as those related to the child’s entry into school and the possible emergence of emotional and social complexities made more evident during this transitional period, can limit the understanding of the child’s needs and their possible developmental difficulties, as well as undermine the process of individuation and their autonomy and independence, leading to an increase in both internalizing and externalizing problems. This rigidity may be related to difficulties in dealing with a gifted child and their needs, feelings of inadequacy, and a tendency to be less compliant while displaying an authoritarian style [34,42], which in turn may influence psychological wellbeing and contribute to individual relational problems [20,43,44].

Furthermore, rigid and extremely demanding parents may not be able to support the emotional complexity of their children, who may find it difficult to manage their own emotional experiences to the point of exhibiting internalizing and externalizing problems. Moreover, the school system can fail to support children with adjustment problems, which can further affect their socio-emotional wellbeing [45]. In this context, externalizing problems can also be seen as a form of protest and an attempt to signal unsatisfied needs. These results are particularly meaningful considering that we controlled for IQ levels, which have been associated with adjustment problems in previous studies [22,23].

In the present study, no other unbalanced dimensions of cohesion and adaptability seem to exert an effect on externalizing problems among gifted children, although future studies are needed to deepen our preliminary findings.

The results that emerged in the current study point out the role of rigidity in internalizing and externalizing problems in gifted children, highlighting the role of environmental variables in the adjustment difficulties that this population may experience. While the international literature has consistently highlighted the role of cohesion in children’s adjustment problems and more conflicting results emerged on the role of adaptability [26,31], data found in the present sample, characterized by families with gifted children, seem to suggest the central role of flexibility in gifted child’s internalizing and externalizing problems. This may be due to the specific population studied, in which poor management of change and complexity can affect the wellbeing of the family system and the child’s ability to adapt, especially during transitional periods in the family life cycle, such as entry into school, which can be a particularly vulnerable time for gifted children and their families, when difficulties manifest themselves at the social and emotional levels as the child leaves the family unit [44,46].

## 5. Limitations and Future Directions

The results of the present study have to be considered in light of some limitations: first, the cross-sectional design does not allow us to draw firm conclusions about the causal direction of the association observed, and longitudinal studies are needed to support our results. Furthermore, the sample is not representative of the Italian population, although it is characterized by a large sample size and heterogeneity at the sociodemographic level. The overrepresentation of male gifted children compared to females in the present sample limits the generalizability of our results but can be explained by the fact that male children are more likely to show externalizing behaviors and act out more than females [42]. Because female children appear to have better adaptive skills and are more compliant (e.g., to school demands), they show fewer problems that decrease requests for an IQ assessment. Several studies have found that males have poorer social skills and more externalizing problems compared to females [20,47].

The generalizability of the results found is further limited by using a sample composed only of mothers and their children (while fathers have not been included in the research) as well as by the use of self-report measures based on mothers’ perceptions. Future studies should also include fathers and refer to assessments of the child’s internalizing and externalizing problems made by individuals outside the family system.

Finally, future studies should assess the independent effect of constitutional/temperamental and family environment variables in order to further shed light on risk factors of adjustment problems in this population.

## 6. Conclusions

The results that emerged in the present study highlight the influence of rigidity in the family system on internalizing and externalizing problems in gifted children. These data, if further supported, seem to suggest the need to develop and implement interventions aimed at increasing flexibility in families with gifted children in order to address the psychological, social, and/or academic complexities that can characterize the experience of this specific population during intense periods of stress and transition, thus reducing the risk of adjustment problems in gifted children. In this sense, services and interventions that accompany parenting in the preschool and school years could promote the wellbeing of gifted children and their families in general.

Without adequate preparation and institutions, gifted individuals, feeling excluded and not understood, may come to conform to their peers or, on the contrary, manifest frustration through both internalizing problems and disruptive behavior. It is necessary to address these problems by working as a network—schools, parents, and institutions—to promote an environment that is supportive of the development of gifted children, which in turn will contribute to the promotion of wellbeing and adaptation.

## Figures and Tables

**Table 1:** The table shows the characteristics of the sample.

	Mean	SD
Children’s age	8.03	1.30
Children’s IQ	134.83	9.04
Mothers’ age ^a^	42.24	4.11
Disengagement	23.04	7.37
Enmeshment	29.02	10.45
Rigidity	31.34	10.14
Chaos	48.53	18.10
Internalizing problems	61.58	8.78
Externalizing problems	58.56	7.80
		
	***N***	***%***
Children’s Sex		
Male	297	82
Female	65	18
Age Children		
6 years old	54	14.9
7 years old	77	21.3
8 years old	96	26.5
9 years old	73	20.2
10 years old	62	17.1
Children’s classroom ^b^		
First primary	59	16.3
Second primary	85	23.5
Third primary	95	26.2
Fourth primary	66	18.2
Fifth primary	46	12.7
Mothers’ educational degree ^c^		
Lower secondary degree	8	2.2
Upper secondary degree	50	13.8
University degree	110	30.4
Master post degree	47	13
Family’s geographical origin ^d^		
North Italy	275	75.97
Center Italy	27	7.45
South Italy	29	8.01

Note: *N* = 362. ^a^ 14 missing values; ^b^ 9 missing values. ^c^ 147 missing value. ^d^ 31 missing value.

**Table 2:** The table shows sex differences in relation to gifted child IQ.

	Males	Females	*t*	*p*	Cohen’s *d*		
	M	SD	M	SD			
IQ	135.05	9.15	133.83	8.54	1.03	0.304	0.135

**Table 3:** The table shows bivariate correlations between study variables.

	1	2	3	4	5	6	7	8	9
1. Children’s sex	—								
2. Children’s age	−0.02	—							
	(0.658)								
3. IQ	−0.05	0.06	—						
	(0.401)	(295)							
4. Internalizing problems	−0.07	0.01	−0.06	—					
	(0.195)	(0.983)	(0.292)						
5. Externalizing problems	−0.07	−0.07	−0.12	0.46	—				
	(0.211)	(0.211)	(0.038) *	(0.001) ***					
6. Disengagement	0.04	−0.03	−0.04	−0.04	−0.05	—			
	(0.459)	(0.648)	(0.503)	(0.440)	(0.372)				
7. Enmeshment	0.07	0.01	−0.05	−0.10	0.01	0.27	—		
	(0.240)	(0.916)	(0.434)	(0.070)	(0.910)	(0.001) ***			
8.Rigidity	−0.01	0.09	−0.02	0.17	0.26	0.03	−0.04	—	
	(0.853)	(0.131)	(0.756)	(0.003) **	(0.001) ***	(0.754)	(0.454)		
9. Chaos	0.09	0.01	−0.11	−0.15	−0.01	0.17	0.33	−0.19	—
	(0.135)	(0.997)	(0.058)	(0.011) *	(0.987)	(0.003) **	(0.001) ***	(0.001) ***	

Note = *** *p* < 0.001; ** *p* < 0.01; * *p* < 0.05.

**Table 4:** The table shows the results of the multiple linear regression model predicting internalizing problems.

				95%CI		
Variables	B	SE	b	LL	UL	*p*
Disengagement	−0.02	0.07	−0.02	−0.15	0.11	0.775
Enmeshment	−0.05	0.05	−0.06	−0.14	0.05	0.319
Rigidity	0.12	0.05	0.15	0.03	0.21	0.012 *
Chaos	−0.05	0.03	−0.10	−0.10	0.01	0.106
Child’s IQ	−0.08	0.05	−0.08	−0.18	0.03	0.145
Child’s age	0.06	0.36	0.01	−0.65	0.77	0.871
Child’sex	−1.18	1.21	−0.06	−3.55	1.20	0.330

Note: * *p* < 0.05.

**Table 5:** The table shows the results of the multiple linear regression model predicting externalizing problems.

				95%CI		
Variable	B	SE	b	LL	UL	*p*
Disengagement	−0.08	0.06	−0.08	−0.19	0.04	0.172
Enmeshment	0.02	0.04	0.02	−0.07	0.10	0.705
Rigidity	0.20	0.04	0.28	0.12	0.29	<0.001 ***
Chaos	0.02	0.03	0.05	−0.03	0.07	0.383
Child’s IQ	−0.09	0.05	−0.11	−0.18	0.01	0.053
Child’s age	−0.45	0.32	−0.08	−1.09	0.18	0.162
Child’sex	−1.37	1.08	−0.07	−3.51	0.76	0.207

Note: *** *p* < 0.001.

## Data Availability

Data will be made available upon request.
